# Genetic underpinning of the comorbidity between type 2 diabetes and osteoarthritis

**DOI:** 10.1016/j.ajhg.2023.06.010

**Published:** 2023-07-10

**Authors:** Ana Luiza Arruda, April Hartley, Georgia Katsoula, George Davey Smith, Andrew P. Morris, Eleftheria Zeggini

**Affiliations:** 1 Institute of Translational Genomics, Helmholtz Zentrum München – German Research Center for Environmental Health, 85764 Neuherberg, Germany; 2 Munich School of Data Science, Helmholtz Zentrum München – German Research Center for Environmental Health, 85764 Neuherberg, Germany; 3 TUM School of Medicine, Technical University Munich and Klinikum Rechts der Isar, 81675 Munich, Germany; 4 Technical University of Munich (TUM), School of Medicine, Graduate School of Experimental Medicine, 81675 Munich, Germany; 5 MRC Integrative Epidemiology Unit, University of Bristol, BS8 2BN Bristol, UK; 6 Centre for Genetics and Genomics Versus Arthritis, Centre for Musculoskeletal Research, The University of Manchester, M13 9PT Manchester, UK

**Keywords:** type 2 diabetes, osteoarthritis, multimorbidity, statistical genetics, colocalization analysis, Mendelian randomization

## Abstract

Multimorbidity is a rising public health challenge with important implications for health management and policy. The most common multimorbidity pattern is the combination of cardiometabolic and osteoarticular diseases. Here, we study the genetic underpinning of the comorbidity between type 2 diabetes and osteoarthritis. We find genome-wide genetic correlation between the two diseases and robust evidence for association-signal colocalization at 18 genomic regions. We integrate multi-omics and functional information to resolve the colocalizing signals and identify high-confidence effector genes, including *FTO* and *IRX3*, which provide proof-of-concept insights into the epidemiologic link between obesity and both diseases. We find enrichment for lipid metabolism and skeletal formation pathways for signals underpinning the knee and hip osteoarthritis comorbidities with type 2 diabetes, respectively. Causal inference analysis identifies complex effects of tissue-specific gene expression on comorbidity outcomes. Our findings provide insights into the biological basis for the type 2 diabetes-osteoarthritis disease co-occurrence.

## Teaser

Multimorbidity is a rising public health challenge. Here, we study the genetic underpinning of the comorbidity between type 2 diabetes and osteoarthritis and provide insights into its biological basis. The approach can be applied to any pair of co-occurring diseases.

## Introduction

Multimorbidity is defined as the coexistence of multiple chronic diseases in a single individual.^1^ Worldwide, over 50% of the population older than 65 years is affected by more than one long-term medical condition simultaneously.^2^ Commensurate with the rise in life expectancy and average population age, multimorbidity is an increasing global health challenge. However, the majority of health and drug development research is focused on treating and/or preventing individual diseases, leading to interventions that are currently not optimally designed to assist individuals suffering from multiple health conditions.

The most prevalent multimorbidity pattern among women and men is the combination of cardiometabolic and osteoarticular diseases,^3^ exemplified by the highly prevalent co-occurrence of type 2 diabetes and osteoarthritis.^4^ Between 2009 and 2016, approximately one in three adults with prediabetes in the US suffered from arthritis.^5^ Osteoarthritis is the most common whole-joint chronic disorder, affecting over 520 million people worldwide.^6^ It is a degenerative disorder characterized by a local and systemic low-grade inflammation state, irreversible loss of cartilage, and additional bone formation that results in pain, its most prevalent symptom.^7^ Across the globe, type 2 diabetes affects over 430 million people and is characterized by elevated blood glucose levels and insulin resistance.^6^ Both osteoarthritis and type 2 diabetes are complex diseases influenced by genetic, demographic, and lifestyle factors, such as older age and obesity.^8^

The majority of observational studies have reported a positive epidemiological association between type 2 diabetes and osteoarthritis of the hip or knee.^4^ In a meta-analysis including 1,040,175 individuals, the unadjusted odds ratio (OR) for type 2 diabetes in individuals with osteoarthritis versus non-osteoarthritis was 1.41 (95% confidence interval [CI] = [1.21, 1.65]).^9^ For individuals with type 2 diabetes, the overall risk of osteoarthritis was also higher than for individuals without type 2 diabetes (unadjusted OR = 1.46, 95% CI = [1.08, 1.96], n = 32,137).^9^ Articular joint-specific analyses have shown a stronger link between type 2 diabetes and knee osteoarthritis than hip osteoarthritis.^9^

Mendelian randomization (MR) analyses^10^ suggest no causal relation between liability to type 2 diabetes and knee osteoarthritis,^11^ whereas body-mass index (BMI) has been shown to be causal for both diseases.^12^^,^^13^ When adjusting for BMI, studies linking type 2 diabetes and osteoarthritis have yielded conflicting results.^4^^,^^9^^,^^14^ Considering that obesity is a major risk factor for both diseases studied here, genetic variants associated with different physiological characteristics of increased adiposity are expected to be shared risk variants for the comorbidity. However, those variants could exert their effects on the comorbidity through alternative biological pathways to obesity through horizontal pleiotropy.^10^

Given the increase of the world’s elderly population and the chronic nature of this highly prevalent pair of diseases, understanding their shared genetic background is important. Here, we focus on disentangling shared genetic risk loci between type 2 diabetes and osteoarthritis, including integration with functional genomics data in relevant cell types, in order to identify effector genes and provide insights into common underpinning mechanisms of disease development.

## Material and methods

### Datasets

For osteoarthritis, we used a recent large genome-wide association study (GWAS) meta-analysis from the Genetics of Osteoarthritis (GO) consortium.^15^ In total, it comprises data from 826,690 individuals (177,517 affected individuals) from mostly white European ancestry for 11 different osteoarthritis phenotypes. In this study, we used the following osteoarthritis phenotypes: knee, hip, knee and/or hip, total knee replacement (TKR), total hip replacement (THR), total joint replacement (TJR), and osteoarthritis at any site (all). An overview of the number of affected individuals, control individuals, and the total individuals for each study can be found in Table 1. For type 2 diabetes, the GWAS meta-analysis unadjusted for BMI from the DIAMANTE consortium was used.^16^ It includes data from 898,130 individuals (74,124 affected individuals) of European ancestry.

**Table 1:** Sample sizes of GWASs used in this study

**OA phenotype**	**Affected individuals**	**Control individuals**	**Total**
All	177,517	649,173	826,690
Knee	62,497	333,557	396,054
Knee and/or hip	89,741	400,604	490,345
Hip	36,445	316,943	353,388
TKR	18,200	233,841	252,041
TJR	40,887	327,689	368,576
THR	23,021	296,016	319,037

An overview of the osteoarthritis (OA) phenotypes used in this work and the number of affected individuals, control individuals, and total number of individuals included in the corresponding GWAS (total knee replacement [TKR], total hip replacement [THR], total joint replacement [TJR], and osteoarthritis at any site [all]).

We also employed molecular quantitative trait locus (QTL) data from disease-specific tissues. For osteoarthritis, we used expression quantitative trait locus (eQTL) data from intact cartilage (n = 95), degenerated cartilage (n = 87), and synovium (n = 77), as well as protein abundance quantitative trait locus (pQTL) data from intact and degenerated cartilage (n = 99).^17^ All samples were collected from individuals with osteoarthritis. For type 2 diabetes, we used eQTL data from pancreatic islets from the InsPIRE consortium.^18^ In the pancreatic islets dataset, 37 individuals out of 420 were diabetic.

We aligned the effect alleles of all datasets used in this paper by inverting the sign of the effect sizes when a mismatch was detected. Chromosome X was not included in any analysis. All datasets used the Genome Reference Consortium Human Build 37 (GRCh37) assembly.

### Measures of adiposity

We used four measures of adiposity: BMI, waist-to-hip ratio (WHR) unadjusted for BMI, whole-body fat mass, and body fat percentage. For BMI (n = 806,834) and WHR (n = 697,734), we used the recent meta-analysis combining data from the GIANT consortium and the UK biobank.^19^ The inverse rank-normalized GWAS summary statistics for whole-body fat mass (n = 330,762) and body fat percentage (n = 331,117) were taken from the Neale’s Lab website (http://www.nealelab.is/uk-biobank/). For each adiposity phenotype, we looked up the effect direction and significance of all variants in the 95% credible set of the colocalized regions between type 2 diabetes and osteoarthritis (Table S11).

### Quantification and statistical analysis

#### Genetic overlap of type 2 diabetes and osteoarthritis phenotypes

We conducted a linkage disequilibrium (LD) score regression analysis using the LDSC software (v.1.0.1) with --rg flag to estimate the genetic correlation between each osteoarthritis phenotype and type 2 diabetes (Table S1).^20^ Because the majority of the GWASs used here comprise data of European ancestry individuals only, pre-computed LD scores from the 1000 Genomes European ancestry haplotypes were used.^21^ To assess the potential for chance findings when performing multiple statistical analyses, we performed a permutation-based analysis. We randomly permuted the effects (*Z* scores) of the variants for the osteoarthritis phenotypes 10,000 times while fixing the effects for type 2 diabetes. Running LD score regression on each permuted dataset yielded an empirical p value for the genetic correlation of type 2 diabetes and each analyzed osteoarthritis phenotype.

#### Statistical colocalization analysis

We defined regions of 2 Mb (±1 Mb) around established independent association signals from each disease. For type 2 diabetes, we selected all primary and secondary independent signals from the BMI-unadjusted GWAS (p value threshold = $5\times 10^{-8}$). For osteoarthritis, we selected the risk signals for the respective phenotype at the adjusted genome-wide significance of $1.3\times 10^{-8}$. For each osteoarthritis phenotype, we performed regional pairwise statistical colocalization analysis with type 2 diabetes using the *coloc.abf* function from the *coloc* R package (version 3.2.1).^22^ Colocalization analyses were conducted using estimated regression coefficients (effect sizes) and standard errors (Table S2). In short, this function calculates posterior probabilities for five association configurations under the assumption of a single causal variant per trait. These configurations are summarized in the hypotheses below:

• H0: no trait has a genetic association in the region.
• H1: trait 1 has a genetic association in the region.
• H2: trait 2 has a genetic association in the region.
• H3: both traits have a genetic association in the region but with different causal variants.
• H4: both traits share a genetic association (single causal variant) in the region.

For all osteoarthritis phenotypes, we used the default prior probabilities of the *coloc* R package. We considered evidence for colocalization if the posterior probability of H4 (PP4) > 0.8. For each genomic locus of colocalization, we calculated a 95% credible set for the causal variant by taking the cumulative sum of the variants’ posterior probabilities to be causal conditional on H4 being true. LD between the single-nucleotide polymorphisms (SNPs) was calculated using plink (v.2.0 alpha)^23^ based on the UK biobank^24^ and was used for visualizing the results in regional association plots.

#### Knockout mouse phenotypes

We performed a schematic search for each gene in the vicinity of the genomic loci that colocalize between type 2 diabetes and osteoarthritis to screen for knockout mice showing phenotypes related to type 2 diabetes or osteoarthritis. The databases used in this scope were the International Mouse Phenotyping Consortium (IMPC) (https://www.mousephenotype.org/), Mouse Genome Informatics (MGI) (http://www.informatics.jax.org/), and Rat Genome Database (RGD) (https://rgd.mcw.edu/). For IMPC and RGD, we extracted the knockout mice phenotypes for each potential effector gene using the programmatic data access via their application programming interface (API). For MGI, we used the MGI batch query.

For type 2 diabetes, we looked for insulin- and diabetes-related phenotypes that included the following terms: insulin, glucose, diabetes, hyperglycemia, pancreas, pancreatic, obesity, BMI, body weight, body mass, body fat, beta cell, and glucosuria. For osteoarthritis, we looked for musculoskeletal phenotypes including the terms skeletal, muscle, bone, osteo, arthritis, muscular, joint, body size, growth, stature, and height.

#### Rare and syndromic human diseases

To investigate whether any analyzed genes are associated with a monogenic disorder, we extracted data from the Online Mendelian Inheritance in Man (OMIM) (https://omim.org/) by using their API. The terms we looked up for osteoarthritis-related phenotypes were bone, muscle, skeleton, osteo, arthritis, muscular, joint, body size, growth, skeletal, stature, height, hand-foot-uterus, synostosis, Martsolf, Warburg, leukodystrophy, squalene, and FINCA (as an abbreviation of fibrosis, neurodegeneration, and cerebral angiomatosis). For type 2 diabetes, we searched for insulin, glycemia, glucose, diabetes, pancreas, pancreatic, obesity, BMI, body weight, body mass, body fat, beta cell, glucosuria, Martsolf, aciduria, Aicardi-Goutières, and FINCA.

#### Differential gene expression

We explored whether the analyzed genes show differential expression for type 2 diabetes and osteoarthritis using published summary statistics from RNA sequencing (RNA-seq) datasets. For osteoarthritis, we assessed differential expression by comparing paired intact and degraded osteoarthritis cartilage from 124 individuals.^25^ Because the samples were collected within person, the data is automatically robust against cofactors such as age and population structure. For type 2 diabetes, we used RNA-seq data from surgical pancreatic tissue samples from metabolically phenotyped pancreatectomized individuals. Samples were collected from 18 non-diabetic individuals and 39 individuals who were previously diagnosed with type 2 diabetes.^26^ The differential expression analysis was based on a linear model with age, sex, and BMI as covariates. We considered genes that changed more than 1.5-fold in either direction and had an adjusted p value < 0.05 to be differentially expressed between degraded (high-grade) and intact (low-grade) osteoarthritis cartilage and diabetic versus healthy pancreatic islets for osteoarthritis and type 2 diabetes, respectively.

#### Multi-trait statistical colocalization analysis with eQTL and pQTL data

First, we superimposed molecular QTL information from disease-specific tissues by performing multi-trait molecular QTL-GWAS colocalization analyses. The analyses were performed only on the variants in the 95% credible set. The input consisted of three summary statistics: one from the type 2 diabetes GWAS, one from the osteoarthritis phenotype GWAS, and one from the disease-relevant tissue molecular QTL dataset. Because one variant is tested for multiple genes in an eQTL dataset or multiple proteins for the pQTL datasets, we performed the colocalization gene-wise or protein-wise, respectively, such that for each analysis a single molecular QTL summary statistic is available for each variant. If the 95% credible set consisted only of a single variant for each gene or protein, we included all variants in a 1-Mb window in the analysis.

For the multi-trait statistical colocalization analyses, we used the R package *HyPrColoc* (v.1.0.0).^27^ We conducted regional gene-wise analysis to assess whether all traits colocalize by switching off the Bayesian divisive clustering algorithm (bb.alg = FALSE). In a similar manner to the *coloc* package, we used *HyPrColoc* to estimate the posterior probabilities and identify candidate effector genes using multiple traits as input. For consistency, evidence for colocalization was considered at a threshold of 0.8 for PP4. The type 2 diabetes and osteoarthritis GWAS meta-analyses share five cohorts. Although the samples overlap, we assumed independence between the datasets, as instructed by the developers of the *HyPrColoc* package.

Using the prior knowledge that type 2 diabetes and osteoarthritis colocalize in the analyzed genomic loci, we adapted the prior parameters of the *HyPrColoc* algorithm accordingly. The first parameter, *prior.1*, which denotes the probability that a SNP is associated with one trait only, was set to 1 × 10^−10^, six times smaller than the default. We set the second parameter *prior.2* to 0.7 instead of the default of 0.98. 1-*prior.2* denotes the prior probability of a SNP being associated with an additional trait and 1-(*prior.2*)^2^ with the SNP being associated with the two other traits. LD between SNPs was again calculated using plink (v.2.0 alpha)^23^ based on the UK biobank.^24^

#### Scoring of potential effector genes

In genomic loci that colocalized between type 2 diabetes and at least one osteoarthritis phenotype with a PP4 > 0.8, we analyzed all genes in a 1-Mb window on either side of the lead variant of the 95% credible set. We incorporated orthogonal multi-omics and functional information to derive a list of high-confidence effector genes for the type 2 diabetes-osteoarthritis comorbidity.

Except for the pQTL analysis, all four above-mentioned biological lines of evidence were tested for both osteoarthritis and type 2 diabetes, yielding one separate score for each disease. Additionally, we incorporated information about previously established high-confidence effector genes for the individual diseases. For type 2 diabetes, we defined genes as high confidence if their top score in the type 2 diabetes knowledge portal was at least 4 (https://t2d.hugeamp.org/). For osteoarthritis, we selected genes scored as high confidence by the GO consortium.^15^ Because our analysis overlaps with criteria used to define a gene as high confidence for the individual diseases, we followed an approach to incorporate this information orthogonally: if a gene is high confidence for a disease but scored zero in our analysis, we updated the respective disease score to one.

We also looked up all variants in the 95% credible sets and searched for any missense variants for the genes located in the colocalized genomic loci. The results of this lookup were consolidated into an additional score for each gene, defined as the missense variant score. The total score was defined as the sum of the osteoarthritis score, the type 2 diabetes score, and the missense variant lookup. However, if for a gene only the missense variant score is non-zero, the total score was set to zero because it is not relevant for the type 2 diabetes-osteoarthritis comorbidity.

Based on the scoring of the six orthogonal biological lines of evidence, we defined genes as potential effector genes if they showed at least one line of evidence for either one of the diseases. Genes that scored at least one line of evidence for osteoarthritis and one for type 2 diabetes were defined as likely effector genes for comorbidity. High-confidence effector genes were a subset of the likely effector genes that scored at least 3 in the total score (Table S3).

To further analyze our set of effector genes, we grouped them according to the osteoarthritis localization. If a gene is located in a genomic locus that colocalizes only between type 2 diabetes and the following three osteoarthritis phenotypes (osteoarthritis at any site, knee and/or hip osteoarthritis, and/or TJR), then it was considered to be associated equally with knee and hip osteoarthritis. If, in addition, the genomic locus colocalizes between type 2 diabetes and knee and/or TKR, then we considered the gene to be mostly associated with knee osteoarthritis. Similarly, if it also colocalizes between type 2 diabetes and hip and/or THR, then the gene was classified as mostly related to hip osteoarthritis.

#### Multi-trait statistical colocalization analysis with adiposity measures

In the genomic regions that colocalized between type 2 diabetes and osteoarthritis, we performed multi-trait colocalization analyses between type 2 diabetes, osteoarthritis, and the above-mentioned measures of adiposity. The analyses were performed only on the variants in the 95% credible set. If the 95% credible set consisted only of a single variant, for each gene or protein, we included all variants in a 1-Mb window on either side of the single variant in the analysis. As for the molecular QTL colocalization, we used the same functions of the R package *HyPrColoc* (v.1.0.0) and adjusted the prior parameters accordingly (*prior.1* = 1 × 10^−10^, *prior.2* = 0.7).^27^ For consistency, evidence for colocalization was considered at a threshold of 0.8 for PP4.

#### Pathway analysis

We performed gene set enrichment analyses on the likely and high-confidence effector genes stratified by knee or hip osteoarthritis association (Table S5). The number of genes in each set is summarized in Table 2. We used the human resources and the enrichment software from the ConsensusPathDB (http://cpdb.molgen.mpg.de/) to examine the functional annotation of each gene set by testing their enrichment among curated networks in humans.^28^ We used the networks from Reactome,^29^ KEGG,^30^ WikiPathways^31^ and Gene Ontology.^32^ For the latter, we included the subcategories molecular function, biological processes, and cellular component up to level 4. We required a minimum overlap of two genes for enrichment. The significance threshold was set at false discovery rate (FDR) < 0.05.

**Table 2:** Number of genes in each gene set

**Gene set**	**Number of genes**
Likely effector genes	72
Likely effector genes related to knee	67
Likely effector genes related to hip	43
HC effector genes	19
HC effector genes related to knee	18
HC effector genes related to hip	10

HC = high confidence

#### Classification of high-confidence genes on the basis of association with obesity

We have classified the high-confidence genes based on the level of association with obesity. For level 1, we searched OMIM for diseases or susceptibility to diseases associated with obesity. For level 2, we conducted a search on PhenoScanner (v.2, http://www.phenoscanner.medschl.cam.ac.uk/)^33^ by querying their database using their R package *phenoscanner* and Ensembl (GRCh37) by using their API in R. If variants in the genes were associated with phenotypes associated with obesity, the gene was defined as level 2. We tried to capture different aspects of obesity by looking for following adiposity phenotypes: BMI, WHR, weight, fat percentage, and fat mass. We combined the results of PhenoScanner and Ensembl to maximize the genes associated with adiposity measures. The remaining genes were included in level 3 and were defined as having no association with obesity.

#### Druggable genome

To outline drug repurposing targets, we queried the druggability status of the 72 likely effector genes for the comorbidity. We used the Druggable Genome database, which consists of 4,479 genes that are classified into three tiers depending on their progress in the drug development pipeline.^34^ Tier 1 included 1,427 genes that are clinical-phase drug candidates or targets of already-approved small molecules and biotherapeutic drugs. Tier 2 consisted of 682 genes that encode targets with known bioactive drug-like small-molecule binding partners and genes with ≥ 50% identity (over 75% of the sequence) with approved drug targets. Tier 3 comprised 2,370 genes encoding secreted or extracellular proteins, proteins with more distant similarity to approved drug targets, and members of key druggable gene families that were not included in tier 1 or 2. Tier 3 was further subdivided to prioritize genes in proximity (±50 kbp) to a GWAS SNP from the GWAS catalog^35^ and had an extracellular location (Tier 3A). Tier 3B is composed of the remaining genes.

For the likely effector genes included in tier 1, we further examined the approved or in-clinical-trial drugs by using the DrugBank online database (https://www.drugbank.com, accessed on August 1, 2022).

#### Causal inference analysis

Causal inference was strengthened through use of bidirectional two-sample MR between type 2 diabetes and all analyzed osteoarthritis phenotypes.^10^ We used the *TwoSampleMR* R package (v.0.5.6), which is curated by MR-Base.^36^ We performed causal inference analyses on the full summary statistics (Table S6). For all analyses, instrumental variables (IVs) were selected as the genome-wide significant (p value ≤ $5x10^{-8}$) and independent SNPs from the full data. Independence was defined as LD-based clumped SNPs with a strict LD threshold of $R^{2}=0.001$ over a 10-Mb window on either side of the index variant. To assure that the IVs are more strongly related to the exposure than to the outcome, we applied Steiger filtering.^10^ We applied the inverse-variance weighted (IVW) method, which performs a random-effects meta-analysis of the Wald ratios for each SNP, and the weighted median (WM) method. Finally, we performed sensitivity analyses by testing for heterogeneity based on the Q statistic using the *mr_heterogeneity* function from the *TwoSampleMR* R package. Horizontal pleiotropy was assessed through the intercept of the MR-Egger regression.^37^ To account for multiple testing, p values were adjusted via the FDR approach.^10^

#### Two-step MR

We performed a two-step MR analysis between different adiposity measures and type 2 diabetes or osteoarthritis using *cis* eQTLs of each high-confidence effector gene in disease-relevant tissues as mediators (Table S7).^38^ In the first step, we assessed whether adiposity was causal for the expression of our genes in the respective analyzed tissues. To assure independence of IVs between the two steps, we excluded independent eQTLs from the risk variants of each adiposity measure. Independence was defined by local LD-based clumping with $R^{2}=0.001$ over a 10-Mb window on either side of the index variant.

For the second step, we used independent genetics variants associated with the expression of each high-confidence gene as IVs and conducted a two-sample MR analysis between each of our genes and type 2 diabetes or osteoarthritis. For each analyzed tissue and each gene-disease pair, we conducted one MR analysis using the *TwoSampleMR* R package (v.0.5.6).^36^ If only one SNP was available after clumping and harmonizing the data, we employed the Wald ratio method. If more than one SNP remained after the pre-processing steps, we applied the IVW method and tested for heterogeneity with the *mr_heterogeneity* function. Moreover, if more than three SNPs were used for the causal inference analysis, we also tested for horizontal pleiotropy through MR-Egger regression.^37^ Additionally, we estimated the F statistics from summary-level data as ${mean(beta}^{2}/{se}^{2})$ to assess the strength of the IVs.^10^ Finally, we adjusted the p values for multiple testing by using the FDR approach.

#### Tissue-specific effects

We determined the tissue-specific role of BMI in both osteoarthritis and type 2 diabetes using MR restricted to BMI instruments colocalizing with eQTLs in brain and adipose tissue, respectively, as described in Leyden et al.^39^ (Table S12). In brief, summary-level MR was performed restricted to the 86 adipose tissue-colocalizing SNPs and the 140 brain tissue-colocalizing SNPs, where the numerator of the Wald ratio is the SNP effect on osteoarthritis or type 2 diabetes and the denominator is the effect estimate for the SNP on BMI from a GWAS meta-analysis of UK Biobank and the GIANT consortium^40^ available at the MR-Base platform.^36^ Osteoarthritis summary statistics were extracted from the recent GWAS of hip and knee osteoarthritis from the GO consortium.^15^ Type 2 diabetes summary statistics were extracted from the recent European DIAMANTE consortium GWAS.^16^

We used IVW meta-analysis of the individual SNP Wald ratios to estimate the causal effects of adipose-tissue-instrumented BMI and brain tissue-instrumented BMI on each outcome. As sensitivity analyses, we performed MR-Egger^37^ to determine the potential role of pleiotropic effects (i.e., mediated via BMI-independent pathways), which gives a pleiotropy robust estimate of the causal effect assuming that there is no correlation between instrument strength (i.e., the association of the SNP with BMI) and the pleiotropic effect.^10^ We also performed WM analysis, which gives an unbiased estimate of the causal effect as long as less than 50% of the SNPs are invalid instruments.^41^ We performed a *Z* test to assess the effect difference between adipose tissue- and brain tissue-instrumented BMI MR analyses.

## Results

### Insights into disease biology and treatment targets

**Figure 1:**
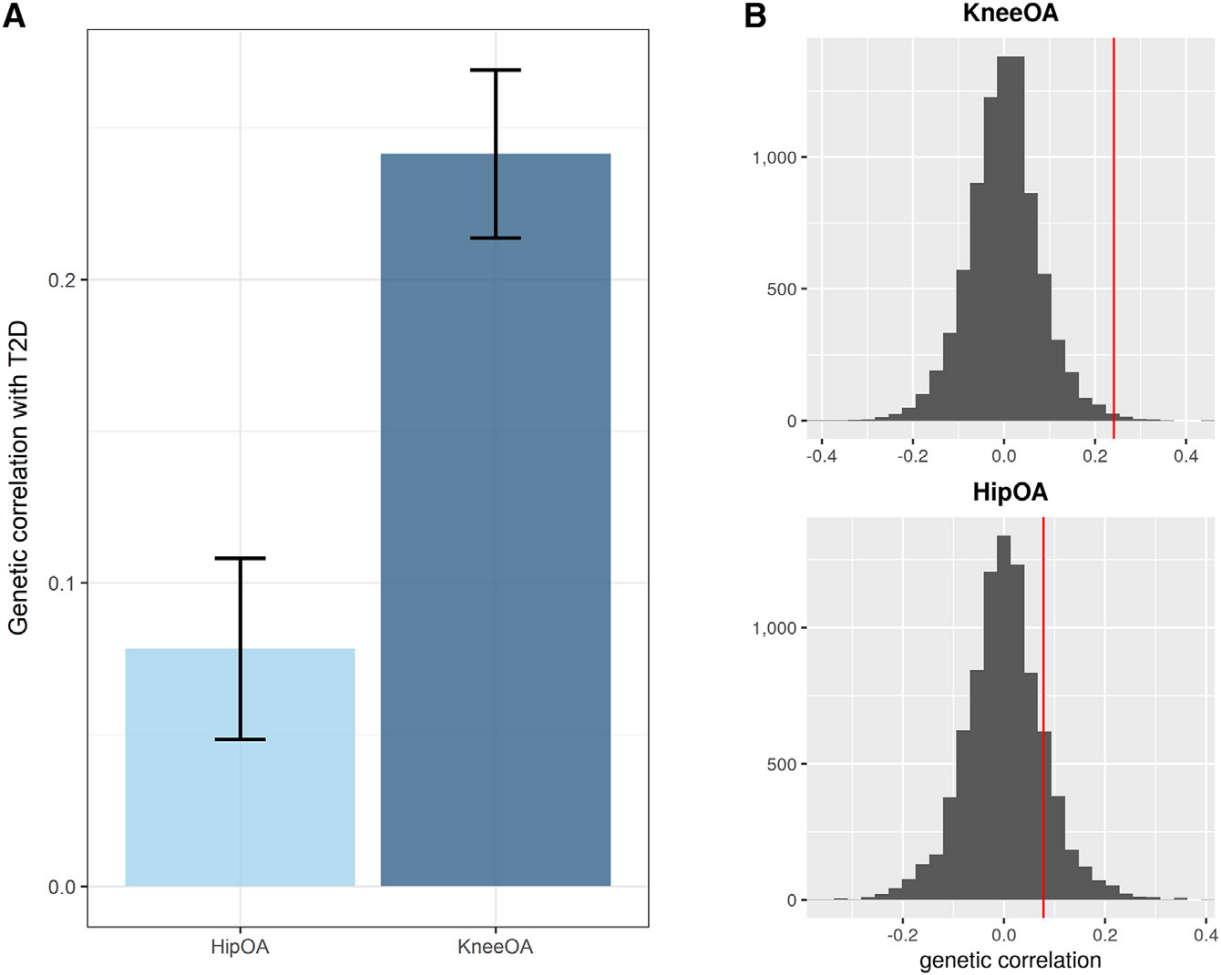
Stronger evidence for a genetic correlation between type 2 diabetes and knee osteoarthritis than between type 2 diabetes and hip osteoarthritis (A) Genetic correlation (r_g_) results between type 2 diabetes (T2D) and knee or hip osteoarthritis (OA). The error bars represent the standard error of the estimated genetic correlation. (B) Permutation-based testing results for knee OA and hip OA, respectively. The red line is the actual correlation.

We first assessed the genetic correlation between type 2 diabetes ($N_{cases}=74,124$, $N_{controls}=824,006$) and osteoarthritis (knee: $N_{cases}=62,497$, $N_{controls}=333,557$; hip: $N_{cases}=36,445$, $N_{controls}=316,943$) on a genome-wide scale by using data from the recent large GWAS meta-analyses (Table S1 and Figure 1). In line with epidemiological evidence, we found a greater magnitude of genetic correlation between type 2 diabetes and knee osteoarthritis (r_g_ = 0.241, SE = 0.028, p = 2.65 × 10^−18^) than osteoarthritis of the hip (r_g_ = 0.078, SE = 0.029, p = 0.008) (Figure 1A). To assess the potential for bias due to overlapping samples and different sample sizes, we also performed a permutation-based analysis (empirical p value for knee = 0.005, empirical p value for hip = 0.142) (Figure 1B and Table S2). Causal inference analyses using MR showed evidence for a non-causal relationship between the two diseases (Table S6), consistent with smaller-scale studies in the literature.^12^

**Figure 2:**
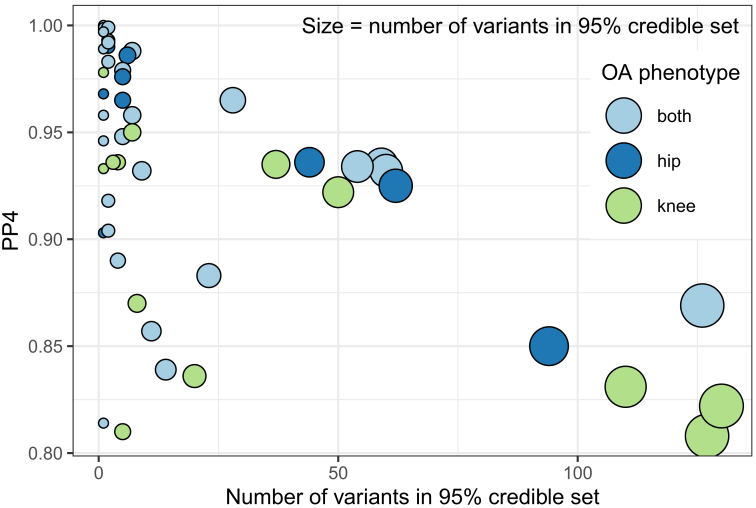
Overview of colocalizing regions between type 2 diabetes and osteoarthritis The y axis depicts the posterior probability of a shared causal variant (PP4) and the x axis the number of variants in the 95% credible set for the causal variant. Each point represents a colocalized signal between type 2 diabetes and one osteoarthritis (OA) phenotype. Point size is proportional to the number of variants in the colocalization analysis 95% credible set. We find strong statistical evidence for colocalization (PP4 > 0.8) for 51 signals. Some of those 51 signals colocalize between type 2 diabetes and more than one OA phenotype, resulting in 18 unique colocalizing genomic loci.

Using pairwise Bayesian colocalization analyses on genome-wide significant regions for osteoarthritis ($p=1.3\times 10^{-8}$) or type 2 diabetes ($p=5\times 10^{-8}$), we found robust evidence of colocalization (posterior probability of a shared causal variant ≥ 0.8) for 51 signals. Some of those signals colocalize between type 2 diabetes, and more than one osteoarthritis phenotype related to the knee and/or hip, resulting in 18 unique colocalizing genomic loci (Table S2 and Figures S5–S22). Ten of those loci colocalize with type 2 diabetes for both hip and knee osteoarthritis, two colocalize for hip osteoarthritis only, and six colocalize only for knee osteoarthritis. In three genomic loci, the 95% credible set for the causal variant from the colocalization analysis consisted of a single variant (Figure 2).

**Figure 3:**
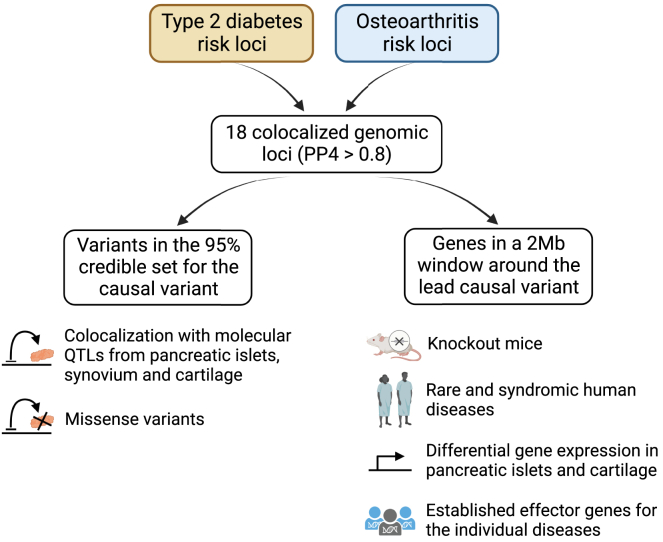
Study design to derive a list of high-confidence effector genes for the type 2 diabetes and osteoarthritis comorbidity In each of the unique 18 genomic loci that colocalized between type 2 diabetes and osteoarthritis with a posterior probability of a single shared causal variant (PP4) ≥ 0.8, we explored all genes in a 1-Mb window on either side of the lead variants of the 95% credible set for the causal variant of the colocalization analysis. For each gene, we searched databases for knockout mice and rare and syndromic human diseases for pre-defined type 2 diabetes- and musculoskeletal-related phenotypes. We also examined differentially expressed genes (DEGs) in pancreatic islets of healthy versus diabetic individuals and of degraded versus intact osteoarthritis cartilage. We also assessed whether the genes were already previously defined as established effector genes for the individual diseases. We examined all variants in the 95% credible set for the causal variant of each colocalization locus for missense variants within genes located in the colocalized genomic loci. We performed regional multi-trait colocalization analyses between type 2 diabetes, each osteoarthritis phenotype, and molecular QTLs from disease-relevant tissues.

To resolve the colocalizing signals between type 2 diabetes and osteoarthritis, we have incorporated multi-omics data and functional information. On the basis of six complementary lines of evidence, we analyzed and scored all 906 genes located in the colocalized genomic loci and identified shared high-confidence candidate effector genes for the type 2 diabetes-osteoarthritis comorbidity (Figure 3 and Table S3). Our analysis included further statistical colocalization of the shared signals with gene eQTLs and pQTLs from disease-relevant tissues (cartilage chondrocytes, synoviocytes, and/or pancreatic beta cells). Twelve of the 18 colocalizing regions between type 2 diabetes and osteoarthritis showed statistical evidence for colocalization with a molecular QTL. We searched the variants in the 95% credible set for any missense variant associated with the candidate genes. As a further line of evidence, we assessed whether the genes were differentially expressed in pancreatic islets from healthy versus diabetic individuals and intact versus degraded osteoarthritis cartilage. We searched the genes in databases for knockout mice and for rare and syndromic human diseases for association with pre-defined phenotypes related to type 2 diabetes and osteoarthritis. Finally, we included information on curated, previously defined effector genes for the individual diseases.

**Figure 4:**
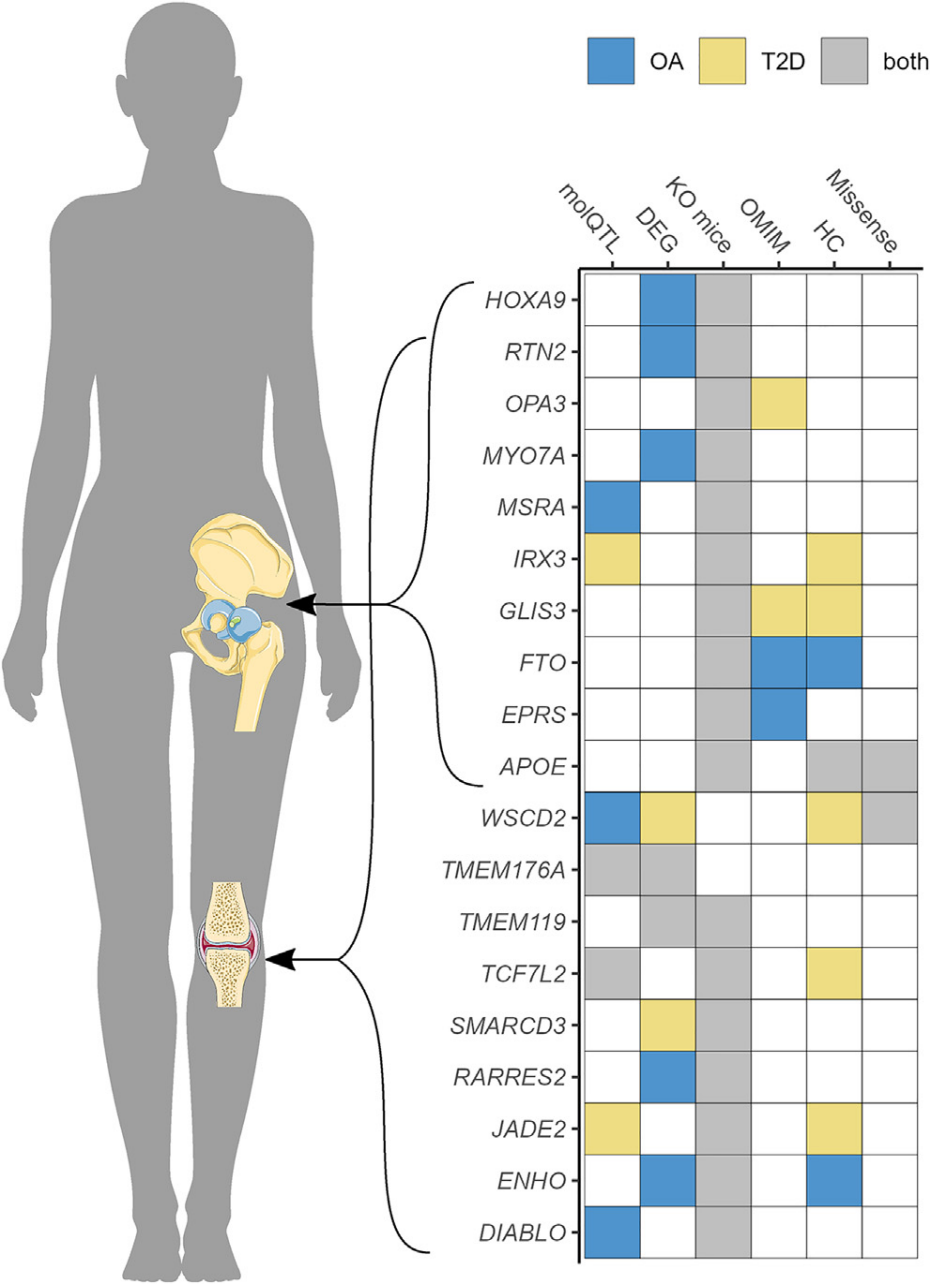
Overview of the 19 high-confidence effector genes for the type 2 diabetes and osteoarthritis comorbidity Genes are stratified based on the joint affected by osteoarthritis. The scoring of the six biological lines of evidence is depicted on the right (material and methods). OA = osteoarthritis; T2D = type 2 diabetes; molQTLs = molecular quantitative trait loci; DEG = differential expressed genes; KO mice = knockout mice; OMIM = Online Mendelian Inheritance in Man; HC = previously defined high-confidence effector genes; missense = missense variant.

We defined 72 genes as likely effector genes for the type 2 diabetes-osteoarthritis comorbidity, as they displayed at least one line of supporting evidence for being involved in both diseases. Of the 72 likely effector genes, 19 showed at least three lines of evidence and were defined as high-confidence effector genes (Figure 4). These represent relevant candidates for further functional and clinical research. Knockout mouse models for 17 out of the 19 high-scoring genes show phenotypes associated with both type 2 diabetes and osteoarthritis, which supports the role of those genes on the comorbidity. Eleven of these have not previously been defined as high-confidence effector genes for either disease based on a recent osteoarthritis study^15^ and the type 2 diabetes knowledge portal (https://t2d.hugeamp.org/). For two of the high-confidence genes, *APOE* and *WSCD2*, the 95% credible set for the causal variant from the colocalization analysis includes missense variants, namely rs429358 (c.466T>C [GenBank: NM_001302688.2] [p.Cys156Arg]) and rs3764002 (c.797C>T [GenBank: NM_014653.4] [p.Thr266Ile]). Six out of 19 high-confidence effector genes are the nearest gene to the lead variant of the respective colocalizing genomic locus: *WSCD2*, *TCF7L2*, *JADE2*, *GLIS3*, *FTO*, and *APOE* (Figure S3).

We searched the druggable genome for the druggability status of the likely effector genes for the type 2 diabetes and osteoarthritis comorbidity.^34^ Sixteen out of 72 genes were included in the druggable genome (Table S4). Of these, six genes are tier 1 druggable targets (*GIPR*, *TPO*, *PAK1*, *SIGMAR1*, *CTSB*, *NOS3*), i.e., they are targets of drugs that have market authorization or are in clinical development. The GIPR agonist tirzepatide was recently approved for the treatment of type 2 diabetes in adults. It has glucose-lowering effects and has been shown to increase insulin sensitivity.^42^ The PAK1 inhibitor fostamatinib has been approved for the treatment of chronic immune thrombocytopenia.^43^ It is also in clinical trial for the treatment of rheumatoid arthritis in order to alleviate the degree of inflammation of the joints.^44^ SIGMAR1 is a target of multiple approved drugs, including pentazocine, which is an analgesic used to treat moderate-to-severe pain. Naltrexone, an antagonist used in opioid overdose that also targets SIGMAR1, is being investigated for treating obesity.^45^^,^^46^ *TPO* encodes the thyroid peroxidase protein, which is the target of several approved drugs for the treatment of hyperthyroidism. One of these, the thyroid hormone dextrothyroxine, has been shown to lower serum levels of cholesterol in humans, but the interventional study has been discontinued due to serious adverse effects.^47^

The 72 likely effector genes were enriched for several metabolic and cellular processes and for lipid localization and storage pathways. Hip osteoarthritis-related likely effector genes were enriched for bone-development pathways and metabolic processes. The 19 high-confidence effector genes were enriched for biological pathways related to diet and obesity (response to caloric restriction and *FTO* obesity variant mechanism) and for regulation of cell differentiation. The high-confidence genes related to hip osteoarthritis were enriched for the *FTO*-obesity-variant-mechanism pathway, regulation of lipid localization, and for a biological pathway related to skeletal formation (proximal/distal pattern formation) (Table S5 and Figure S4). These results provide biological support for the link between obesity and both diseases and for the association between bone development and hip osteoarthritis.^7^

### Disentangling the effect of obesity

Obesity plays a causal role in both type 2 diabetes and osteoarthritis. To explore the role of obesity on the co-occurrence of type 2 diabetes and osteoarthritis, we studied four different measures that capture different aspects of obesity and adiposity: BMI, WHR, whole-body fat mass, and body fat percentage. Sixteen out of the 18 genomic regions that colocalized between type 2 diabetes and osteoarthritis show evidence of association or colocalization (PP4 > 0.8) with at least one adiposity-related trait (Table S11). Four high-confidence effector genes reside in the two genomic regions that do not show any evidence of colocalization or association with the analyzed measures of adiposity: *TMEM176A*, *RARRES2*, *SMARCD3*, and *GLIS3*. These may point to alternative biological mechanisms other than adiposity in the comorbidity between type 2 diabetes and osteoarthritis for these colocalizing signals.

We classified the high-confidence effector genes according to their level of association with obesity. Level 1 was defined as genes with variants directly associated with obesity or susceptibility to obesity in the OMIM database and included only one gene: *FTO*. Level 2 included nine genes for which variants in the gene were associated with the above-mentioned adiposity phenotypes on PhenoScanner or Ensembl. Finally, level 3 consisted of the remaining nine genes, which were associated with obesity on the basis of our analysis (Table S13).

Next, we sought to investigate whether the adiposity measures that capture different aspects of obesity were causally associated with the expression of high-confidence effector genes in disease-relevant tissues. Within the constraints of the available instruments (material and methods), we found evidence of a causal relationship between several adiposity measures and nine high-confidence effector genes (Table S7). For example, we found that all measures of adiposity have a causal effect on higher expression of *IRX3* in synovium or pancreatic islets and on lower expression of *RTN2* in osteoarthritis cartilage. For the high-confidence effector genes located in the two genomic loci that did not show evidence of association or colocalization with adiposity, the direction of effect was not consistent across the different measures employed.

We assessed the causal role of BMI-associated variants with tissue-specific effects, selected based on evidence of their colocalization with brain or subcutaneous adipose tissue eQTLs.^39^ For type 2 diabetes, we replicated previous results and showed that BMI-associated variants influencing genes expressed in brain tissue exert a stronger effect on the disease than adipose tissue-related variants,^39^ although CIs largely overlapped. For knee osteoarthritis, we observed the same trend (Figure S23). For hip osteoarthritis, the results of the causal inference analysis provide evidence for a stronger effect of BMI-associated variants that colocalize with adipose tissue eQTLs than with brain eQTLs (Table S12). Our results suggest a similar biological underpinning of the adiposity effect captured by BMI on type 2 diabetes and knee osteoarthritis but potentially different processes for hip osteoarthritis.

### Insights gained from individual loci

#### *FTO* and *IRX3*

**Figure 5:**
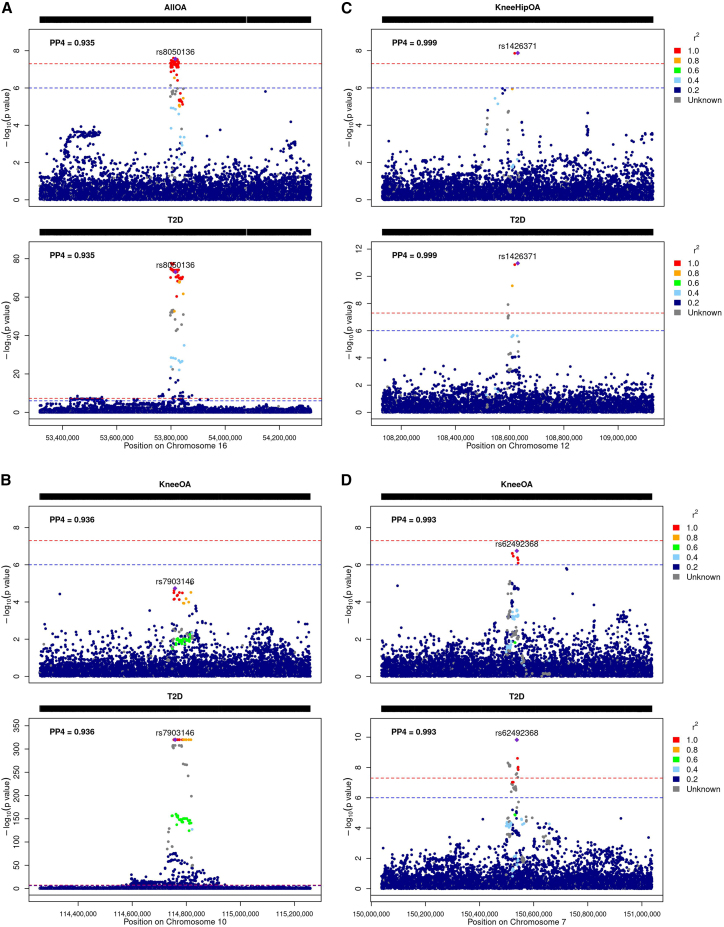
Regional association plots of the highlighted colocalizing regions between type 2 diabetes and osteoarthritis (A–D) *FTO* and *IRX3* region; (B) *TCF7L2* region; (C) *WSCD2* and *TMEM119* region; (D) *TMEM176A* region. The plots are colored on the basis of linkage disequilibrium between the lead causal variant of the colocalization and all other variants in the region. The red dashed line represents the genome-wide significance threshold (p value = 5 × 10^−8^), and the blue dashed line represents a suggestive association threshold (p value = 10^−6^). PP4 = posterior probability of a single shared causal variant; OA = osteoarthritis; T2D = type 2 diabetes; all OA = osteoarthritis at any site.

The obesity-related *FTO* locus colocalizes for type 2 diabetes and osteoarthritis with a posterior probability of a shared causal variant of over 92% (Figure 5A). The 95% credible set from the colocalization analysis consists of multiple variants in high LD with each other. The risk-increasing alleles for the lead causal variants are the same across type 2 diabetes and osteoarthritis. In addition to *FTO*, this locus is associated with a further high-confidence effector gene, *IRX3*. *IRX3* eQTLs in pancreatic islets colocalize with type 2 diabetes and osteoarthritis genetic signals with a PP4 > 0.8.

As shown above, adiposity is causally associated with an increase in *IRX3* expression in pancreatic islets and synovium (Table S7). Here, we performed causal inference analyses between the expression of high-confidence genes at this locus and type 2 diabetes or osteoarthritis. We find evidence for a causal effect of increased expression of *IRX3* in pancreatic islets on increased risk of type 2 diabetes (OR = 1.16, 95% CI = [1.08, 1.25], p value = 4.4 × 10^−5^, F stat = 16.7).

*FTO* is a high-confidence osteoarthritis effector gene involved in skeletal development, adipogenesis, and neuronal function and development.^15^ It is also associated with syndromic human disease growth retardation, developmental delay, and facial dysmorphism (GDFD), which is a lethal autosomal-recessive multiple-congenital-anomaly syndrome.^48^ *IRX3* is a known functional long-range target of *FTO* variants associated with obesity.^49^ *FTO-* and *IRX3*-knockout mice show decreased body weight, decreased bone mineral density, and improved glucose tolerance (high bone mineral density is a risk factor for hip and knee osteoarthritis^50^). We expected genes with an adiposity-driven effect to be involved in the shared genetic etiology of the type 2 diabetes-osteoarthritis comorbidity because obesity constitutes a common risk factor.^8^

#### TCF7L2

*TCF7L2* is one of the highest-scoring effector genes. This genomic locus colocalizes for type 2 diabetes and knee osteoarthritis with a posterior probability of a shared causal variant of over 93% (Figure 5B). Here, the 95% credible set for the causal variant from the colocalization analysis consists of three variants, which have opposite risk-increasing alleles for type 2 diabetes and knee osteoarthritis (Table S8). Variants in *TCF7L2* have not been associated with osteoarthritis at genome-wide significance levels in the recent osteoarthritis GWAS (all variants in the credible set of the colocalization analysis achieve nominal significance in the recent knee osteoarthritis GWAS^15^). Genetic variants associated with *TCF7L2* expression in pancreatic islets and in osteoarthritis cartilage colocalize with this association signal. For the variants in both 95% credible sets from these colocalization analyses, the risk-increasing alleles for type 2 diabetes are associated with a lower BMI, a lower risk of knee osteoarthritis, and an increased expression of *TCF7L2* in pancreatic islets and osteoarthritic cartilage.

We found evidence that increased BMI causes decreased expression of *TCF7L2* in intact and degraded cartilage (Table S7). Additionally, we found evidence that an increase in *TCF7L2* expression in pancreatic islets causes an increase in type 2 diabetes risk (OR = 5.1, 95% CI = [4.7, 5.4], p value < 1 × 10^−300^, F stat = 2,018) and a decrease in knee osteoarthritis risk (OR = 0.83, 95% CI = [0.77, 0.91], p value = 1.77 × 10^−5^, F stat = 18.4). These results are in line with the evidence shown above in support of an opposite effect of the genetic variants associated with the expression of *TCF7L2* in knee osteoarthritis and type 2 diabetes risk.

*TCF7L2* is among the leading signals for type 2 diabetes risk and persists as a top signal after adjustment for BMI.^51^ Our results suggest that *TCF7L2* exerts an effect that goes through an alternative biological pathway to increased BMI. It has been shown that isoforms of *TCF7L2* regulate the expression of genes related to cartilage destruction in human chondrocytes.^52^ *TCF7L2* is a key effector gene of the Wnt/β-catenin signaling pathway. This pathway plays a role in both type 2 diabetes, through glucose homeostasis, and osteoarthritis, through cartilage and bone formation.^53^^,^^54^

#### *TMEM119* and *WSCD2*

Two high-confidence effector genes, *WSCD2* and *TMEM119*, reside in the same genomic locus, which colocalizes for type 2 diabetes and knee osteoarthritis with a posterior probability of 99.9% (Figure 5C). The 95% credible set consists of two variants: rs1426371 and rs3764002, an intronic and a missense variant (amino acid change: Thr266Ile) within *WSCD2*, respectively. The risk-increasing alleles of both variants are concordant for osteoarthritis of the knee and type 2 diabetes. The variant with the highest posterior probability of being causal for osteoarthritis and type 2 diabetes, rs1426371, has reached genome-wide significance levels in the recent knee osteoarthritis GWAS meta-analysis.^15^ The missense variant, rs3764002, is associated with WHR,^55^ type 2 diabetes,^16^ lean mass,^56^ and anxiety and neuroticism.^57^ The missense variant is predicted to alter protein function, and this alteration is predicted to be damaging by both SIFT (https://sift.bii.a-star.edu.sg) and PolyPhen (http://genetics.bwh.harvard.edu/pph2/).

*WSCD2* eQTLs in degraded osteoarthritis cartilage colocalize with type 2 diabetes and knee osteoarthritis with a posterior probability of 99%. The lead eQTLs are rs1426371 and rs3764002. The expression level-increasing alleles are the same as the risk-increasing alleles for both diseases (Table S9). *WSCD2* is also a differentially expressed gene (DEG) in pancreatic islets from individuals with diabetes versus healthy individuals and is downregulated in diabetic islets. Moreover, it has been previously shown that *WSCD2* is functionally associated with type 2 diabetes and positively correlated with insulin secretion.^58^^,^^59^ Conclusions from causal inference analysis were limited as a result of weak instruments (F statistic < 10), which can bias causal effect inference (Table S7). Further research is needed to better understand the biological mechanisms through which *WSCD2* influences the type 2 diabetes-osteoarthritis comorbidity.

*TMEM119* is the second-most highly scoring high-confidence effector gene. It is a DEG in osteoarthritis cartilage and pancreatic islets and is more highly expressed in degraded compared with intact cartilage and in healthy compared with pancreatic islets. Knockout mice for *TMEM119* show phenotypes related to both osteoarthritis and type 2 diabetes, such as decreased body weight, impaired osteoblast differentiation, and decreased compact bone thickness. *TMEM119* is related to bone formation by promoting osteoblast differentiation.^60^ Fewer osteoblasts can lead to a decrease in compact bone thickness, which is also observed in knockout mice.^61^ The overexpression of *TMEM119* in degraded cartilage from individuals with osteoarthritis supports the evidence of an increase in bone formation in later stages of the disease.^62^ However, the lower expression of *TMEM119* in diabetes compared with healthy pancreatic islets points to further potential mechanisms of effect in the comorbidity.

#### TMEM176A

Variants in *TMEM176A*, also a high-confidence effector gene for the investigated comorbidity, have not been previously identified as implicated in either osteoarthritis or type 2 diabetes in the previous recent GWAS for the individual diseases. This locus colocalizes for type 2 diabetes and knee osteoarthritis with a posterior probability of a shared causal variant of 99.3% (Figure 5D). The index variants are rs62492368 and rs7794796, both located in the intron of *AOC1*. rs62492368 is associated with type 2 diabetes,^16^ and rs7794796 is associated with appendicular lean mass.^63^ Type 2 diabetes and osteoarthritis show opposite risk-increasing alleles for all variants in the 95% credible set from the colocalization analysis (Table S10). These variants colocalize with PP4 > 0.8 between the diseases and eQTL data from pancreatic islets and synovium. The index variants from the colocalization with eQTLs also have opposite risk-increasing alleles for both diseases. Similar to the *TCF7L2* locus case, our results suggest that the mechanism through which *TMEM176A* exerts an effect on osteoarthritis and type 2 diabetes may have contrary directions.

We found body fat percentage to be linked to a decrease in the expression of *TMEM176A* in synovium (Table S7), albeit with weak instruments (F statistic < 10). Decreased expression of *TMEM176A* in intact osteoarthritis cartilage and pancreatic islets was associated with reduced risk of type 2 diabetes (pancreatic islets: OR = 1.14, 95% CI = [1.07, 1.21], p value = 8.5 × 10^−5^, F stat = 15.4; cartilage: OR = 1.05, 95% CI = [1.03, 1.08], p value = 8.5 × 10^−5^, F stat = 15.4) and increased risk of knee osteoarthritis (pancreatic islets: OR = 0.93, 95% CI = [0.87, 0.99], p value = 0.048, F stat = 3.9; cartilage: OR = 0.97, 95% CI = [0.94, 0.99], p value = 0.048, F stat = 3.9). This genomic locus is one of the two colocalizing regions that do not show any evidence of statistical colocalization between type 2 diabetes, osteoarthritis, and the adiposity measures studied here. This suggests that this region, and possibly *TMEM176A*, acts through an alternative biological path to adiposity.

Results of the causal inference analysis mirror the output of differential expression analyses conducted in pancreatic islets and osteoarthritic cartilage.^25^^,^^26^ While *TMEM176A* is more highly expressed in pancreatic islets of diabetic individuals than in healthy pancreatic islets, an increase of its expression in the same tissue has a causal effect on increased risk of type 2 diabetes. Similarly, while *TMEM176A* was found to have lower expression in degraded compared with intact cartilage, the reduced expression of this gene in intact cartilage has a causal effect on increased risk of TKR.

## Discussion

We present a genetic databased approach to disentangle the shared genetic etiology between two co-occurring chronic diseases and applied it to a common comorbidity pair: type 2 diabetes and osteoarthritis. Studies have shown a stronger association of BMI with osteoarthritis of the knee than of the hip.^12^ We find stronger statistical evidence of a genetic correlation between type 2 diabetes and knee osteoarthritis than between type 2 diabetes and hip osteoarthritis. By leveraging recent large-scale GWASs for both diseases, we find evidence of colocalization at 18 genomic loci, and by incorporating multi-omics and functional genomics information, we derive a list of 19 high-confidence effector genes for the comorbidity. The majority of genomic loci colocalize for type 2 diabetes and knee, rather than hip, osteoarthritis, in keeping with the genome-wide-correlation analysis results.

Our findings support the epidemiological link between obesity, osteoarthritis, and type 2 diabetes. In this case, only two of the 18 colocalized regions do not colocalize with measures of adiposity. Several of the high-confidence genes, including *FTO* and *IRX3,* are associated with obesity-related traits. We show that the identified high-confidence effector genes are enriched for biological pathways associated with adiposity. Stratifying the high-confidence effector genes into knee or hip osteoarthritis provides further insight into the biological mechanisms underlying the comorbidity. High-confidence effector genes mostly related to hip osteoarthritis are also enriched for biological pathways of skeletal formation, which underlines the strong link between bone development and hip osteoarthritis.^7^ Given that two-thirds of FDA-approved drugs are supported by genetic evidence, we explore the druggable potential of the prioritized genes.^64^ We highlight approved drugs currently used for the treatment of diabetes, obesity, pain, and inflammation.

Observational studies report that the positive association between type 2 diabetes and osteoarthritis persists after adjusting for BMI.^9^ As BMI only captures a limited subset of the effect of adiposity on the comorbidity, this could be a source of residual confounding due to measurement error,^65^ and observed attenuation of association can be underestimated. We performed in-depth analyses to disentangle the role of adiposity on comorbidity and find evidence that *TCF7L2* and *TMEM176A* exert an effect on type 2 diabetes and osteoarthritis through an alternative biological path. Further examination, including functional studies, is needed to dissect the precise way in which these genes affect comorbidity.

Type 2 diabetes and insulin resistance are known to be negatively correlated with bone strength and are also associated with bone fracture.^66^ One possible link between bone and lipid metabolism is the fact that osteoblasts and adipocytes share a common progenitor cell in adult bone marrow with a degree of plasticity that can lead to an imbalance between the two cell lineages.^67^ In support of this link, differentiation regulation of osteoblasts is highlighted by one of the identified high-confidence effector genes, *TMEM119*. In summary, we highlight three potential biological mechanisms underpinning the comorbidity between type 2 diabetes and osteoarthritis: obesity, imbalance between osteoblasts and adipocytes differentiation in adult bone marrow and the Wnt/β-catenin signaling pathway.

We apply statistical colocalization analysis on regions where at least one of the studied diseases shows evidence of genome-wide association (p value < 5 × 10^−8^) but not necessarily both. We have chosen this approach to overcome possible power issues from the individual GWASs due to different disease heterogeneity and sample sizes. Further, we embellish the colocalization results with a deep dive into biological lines of evidence for effector gene involvement in the colocalizing regions. The genetic and functional genomic data employed in this study are biased toward European populations. Going forward, it will be important to expand analyses to data from diverse populations. We have not performed analysis adjusted for BMI or other obesity-related phenotypes to avoid introducing collider bias. The eQTL data from pancreatic islets used in the analyses here comprise almost four times as many samples as the eQTL data from chondrocytes. Therefore, molecular QTL analyses for type 2 diabetes-relevant tissues were better powered than for osteoarthritis-relevant tissues. MR and the subsequent sensitivity analyses were conducted within the constraints of available instruments for expression of the high-confidence genes. This was partially because the molecular QTL data used in this work include *cis* QTLs only, which restricts the analyses to variants within the vicinity of the genes or proteins of interest. Future studies should include a wider array of (as of now unavailable) genome-wide molecular QTLs, including at the single-cell level.

This work provides a proof of concept for the application of a study design that is relevant to any pair of comorbid diseases. We have studied one of the most frequently co-occurring pairs of complex diseases: type 2 diabetes and osteoarthritis. Our findings offer insights into the biological processes underpinning comorbidity and highlight potential drug repurposing opportunities in addition to new targets. As the world population life expectancy continues on an upward trajectory, the challenge of tackling multimorbidity will continue to be high on the healthcare agenda. Genomic-data-based approaches, as exemplified here for type 2 diabetes and osteoarthritis, can help improve our understanding of the co-occurrence of chronic conditions.

## Data Availability

• All original code produced for this manuscript has been deposited at Zenodo and is publicly available (https://doi.org/10.5281/zenodo.7525171). • Any additional information required to reanalyze the data reported in this paper is available from the lead contact upon request, Eleftheria Zeggini.

## Web resources

coloc R package (v.3.2.1) (https://chr1swallace.github.io/coloc/)
ConsensusPathDB-human (http://cpdb.molgen.mpg.de/)
Drugbank (https://www.drugbank.com)
eQTLs and pQTLs for osteoarthritis cartilage and synovium^17^ (https://msk.hugeamp.org/downloads.html)
eQTLs for pancreatic islets^18^ (https://zenodo.org/record/3408356)
Gene Ontology (http://geneontology.org/)
GWAS catalog (https://www.ebi.ac.uk/gwas/)
GWAS summary statistics for BMI and WHR unadjusted for BMI^19^ (https://portals.broadinstitute.org/collaboration/giant/index.php/GIANT_consortium)
GWAS summary statistics for osteoarthritis^15^ (https://msk.hugeamp.org/downloads.html)
GWAS summary statistics for type 2 diabetes^16^ (www.diagram-consortium.org/)
HyPrColoc R package (v.1.0.0) (https://github.com/cnfoley/hyprcoloc)
International Mouse Phenotyping Consortium (https://www.mousephenotype.org/)
KEGG (https://www.genome.jp/kegg/)
LD score regression (v.1.0.1) (https://github.com/bulik/ldsc/)
Mouse Genome Informatics (http://www.informatics.jax.org/)
OMIM database (https://www.omim.org/)
PhenoScanner V2 (http://www.phenoscanner.medschl.cam.ac.uk/)
PLINK 2.0 (https://www.cog-genomics.org/plink/2.0/)
PolyPhen-2 (http://genetics.bwh.harvard.edu/pph2/)
R statistical software (v.4.0.0) (https://www.R-project.org/)
Rat Genome Database (https://rgd.mcw.edu/)
Reactome (https://reactome.org/)
SIFT (https://sift.bii.a-star.edu.sg)
TwoSampleMR R package (v.0.5.6) (https://mrcieu.github.io/TwoSampleMR/)
Type 2 diabetes knowledge portal (https://t2d.hugeamp.org/)
WikiPathways (https://www.wikipathways.org/)

## Supplemental information

Document S1. Figures S1–S23 and Tables S8–S10

Table S1. Genetic correlation matrix for type 2 diabetes and osteoarthritis

Table S2. Overview of colocalized genomic loci between type 2 diabetes and osteoarthritis with additional information about colocalization with molecular QTLs from disease-relevant tissues

Table S3. Overview of scoring of high-confidence effector genes for the type 2 diabetes and osteoarthritis comorbidity

Table S4. Likely effector genes for the type 2 diabetes and osteoarthritis comorbidity included in the druggable genome

Table S5. Results of the pathway analyses of the likely effector genes for the type 2 diabetes-osteoarthritis comorbidity stratified by knee or hip association

Table S6. Steiger-filtered results of bidirectional two-sample Mendelian randomization analyses between type 2 diabetes and osteoarthritis

Table S7. Second step results of two-step Mendelian randomization analysis between gene expression of high-confidence effector genes for the type 2 diabetes and osteoarthritis comorbidity in disease-relevant tissues and either one of the diseases

Table S11. Direction of effect and p value of variants in the 95% credible set of the colocalized regions for the comorbidity

Table S12. Results of two-sample Mendelian randomization analyses between type 2 diabetes or osteoarthritis and BMI risk variants associated with eQTLs in brain and subcutaneous adipose tissue

Table S13. Classification of high-confidence effector genes based on association with obesity

Document S2. Article plus supplemental information
